# Factors Associated with Subsequent Childbirth between Marriage Years in Korea

**DOI:** 10.3390/ijerph182312560

**Published:** 2021-11-29

**Authors:** Jeongok Park, Kyoungjin Lee, Heejung Kim

**Affiliations:** 1 Mo-Im Kim Nursing Research Institute, College of Nursing, Yonsei University, Seoul 03722, Korea; jopark02@yuhs.ac (J.P.); hkim80@yuhs.ac (H.K.); 2 Brain Korea 21 FOUR Project, College of Nursing, Younsei University, Seoul 03722, Korea

**Keywords:** work-life balance, social support, child care, women, working

## Abstract

This study aimed to identify differences in factors associated with subsequent childbirth between the marriage years of 1996–2005 (Group 1) and 2006–2015 (Group 2) using the 2015 National Survey. A total of 5097 eligible participants (2492 and 2605 women in each group, respectively) were included. The main variables consisted of demographic characteristics, socio-economic status, value for child and son, and social support for raising child. For statistical analysis, discrete-time hazard models were used. The common factors associated with subsequent childbirth in both groups were son preference (Group 1: HR = 1.16; 95% CI = 1.06–1.27, Group 2: HR = 1.14; 95% CI = 1.04–1.24) and the favorable value on children (HR = 1.12; 95% CI = 1.01–1.25, HR = 1.11; 95% CI = 1.01–1.22). Only in Group 2, age at the first childbirth (HR = 1.35; 95% CI = 1.31–1.39) and more monthly income (≥4600, <6000: HR = 1.18; 95% CI = 1.04–1.33, ≥6000: HR = 1.15; 95% CI = 1.00–1.32) were significantly associated with subsequent children. Whereas, working women (HR = 0.86; 95% CI = 0.78–0.94) were less likely to have subsequent children. To increase fertility in Korea, the government must provide childcare and deal with factors associated with low fertility considering the reduction in role incomparability for women due to changes in demographic characteristics.

## 1. Introduction

South Korea (hereafter Korea) has one of the lowest fertility rates in the world, and a sharp fall in the fertility rate has been observed since the late-20th century. The total fertility rate in Korea rapidly decreased from 1.74 in 1984 to 1.08 in 2005 and then, surprisingly, fell as low as 0.92 in 2019 [1].

According to previous studies, declining fertility can be caused by demographic, socio-cultural, and economic changes that alter women’s lives [2,3,4]. A contributing factor in demographic changes to fertility is the maternal age at the first birth. Because childbirth has a fixed fertility period, there are biological restrictions involved; therefore, the later the first birth, the lower the chances of having a subsequent childbirth. There is evidence that maternal age at the first birth is associated with subsequent childbirth, which is an essential factor in giving birth to the second baby [4]. From the point of view of socio-cultural and economic changes, higher educational attainment, postponement of marriage, delaying childbearing, change in views on a child, and an increase in economically productive work for women were addressed as the major reasons associated with low fertility. These social changes have been observed in Korea. Since 2005, the college enrollment rate for women (72.7%) has surpassed that of men (65.3%). Moreover, the employment rate of women was 50.8% in 2017, reflecting an increasing trend [5]. Meanwhile, the mean ages of first marriage and childbirth for women were 30.2 and 31.6 years old, respectively, in 2017, and both of them have continuously increased since 1990 [5,6]. Gavin et al. (2007) reported that the decline in Korea’s fertility rate to a very low level since 1995 appears to be entirely a result of the trend in delaying marriage [7]. Socio-cultural and economic status, such as women’s educational level, working status, careers, and income level, are closely related to delaying marriage. Women with higher education are more likely to hold a better job, have higher earnings, and better economic independence. Previous studies reported that these factors have a negative relationship with birth rates [8]. A wider range of life options by obtaining higher education contribute to avoidance or delay in marriage and childrearing, which relieves the burden on women in family obligations formed by marriage [9]. In the same context, postponing having a child could also explain the possibility of choosing to pursue one’s career. Working women with advanced education raising a young child were less likely to have a subsequent child than full-time housewives. The additional caregiver role formed by marriage is a burden for women; therefore, working women tend to consider either forsaking employment or having only one child to reduce the burden of life [10].

The value of children (VOC) can be divided into instrumental and psychological values [11,12]. Instrumental VOC can be defined as an economic and social utility of children, such as providing economic support and succeeding family line, while psychological VOC refers to the emotional benefits of having children, such as providing comfort for parents in their advanced age. Korean married women’s views on children have also changed. The proportion of participants who answered that married couples should have children was 53.8% in 2006 and 46.3% in 2012. Extramarital birth is extremely rare in Korean culture, and it seems that childbearing should be considered in married women.

Blood relatives customarily assist parents in child rearing in all tribal cultures, which are representative of our species’ evolutionary past and therefore of human nature. These relatives include grandparents, older siblings, and others. Research indicates that marital satisfaction is higher in collectivist cultures with a strong extended family than in cultures, such as the US, which are individualistic [13]. Societal measures that facilitate care by grandparents would be expected to encourage parents to have subsequent children, such as financial subsidies or medical help, allowing grandparents to remain in the home. Furthermore, children tend to do better developmentally if cared for by biological kin than by non-kin [14]. They are less likely to be abused and neglected than if raised by a stepparent.

The husband’s housework support and family support are another variable closely related to the fertility rate. Thus, Sear (2017) reported that the interaction with kin was associated with positive fertility in middle- and lower-income groups [15]. 

**Figure 1:**
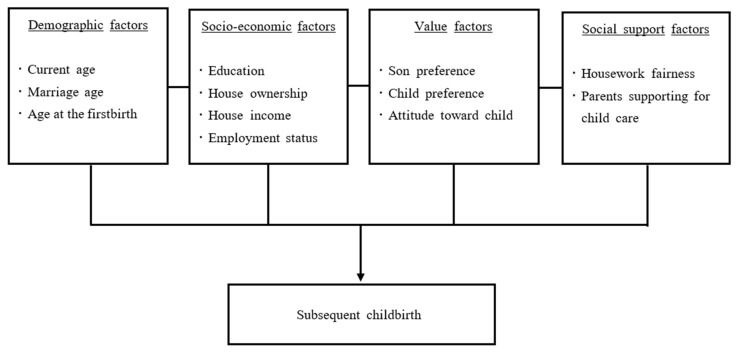
The conceptual framework of the study.

Therefore, the conceptual framework for the current study is developed based on a literature review and included demographics, socio-economics, values, and support factors to examine the differences in factors associated with subsequent childbirth by marriage year (Figure 1). A previous study explored the various factors influencing the low birth rate of married women in Korea [4], but that did not include the Korea Government fertility policy, which active implementation started in 2006. Low fertility in Korea was seen as a national concern in the mid-2000s. The Presidential Commission on Aging Society and Population Policy was established in 2005, and plans for an aging society and population were implemented [16]. Specifically, the first (2006 to 2010) and second (2011 to 2015) five-year plans that encouraged childbirth focused on reducing childcare burdens in married households, for example, by supporting costs of raising and educating children, as well as promoting work-life balance and healthy pregnancy and birth. Despite the Korean government’s 2006 policy to promote childbirth, the total fertility rate has continued to fall from its 2017 level of 1.05 [6]. In order to understand the low fertility in a variety of ways and provide basic data for formulating a new birth encouragement policy, it is critical to examine the differences in factors associated with subsequent childbirth both before and after the plan’s implementation. Therefore, this study aimed to examine differences in factors associated with subsequent childbirth between the marriage years of 1996–2005 and 2006–2015.

## 2. Materials and Methods

This study employed a secondary data analysis using the dataset of the 2015 National Survey on Fertility and Family Health and Welfare (NSFFHW).

### 2.1. Data

The Korea Institute for Health and Social Affairs (KIHASA) conducted the NSFFHW since the early 1970s. The purpose of this primary data collection was to provide baseline evidence supporting the government’s short- and long-term population policies [17]. The 2015 survey was conducted among approximately 11,009 married women of childbearing ages (15 to 49 years old) derived from 12,000 households within 600 survey areas using probability sampling based on the Population and Housing Census in 2010 [17].

### 2.2. Participants

**Figure 2:**
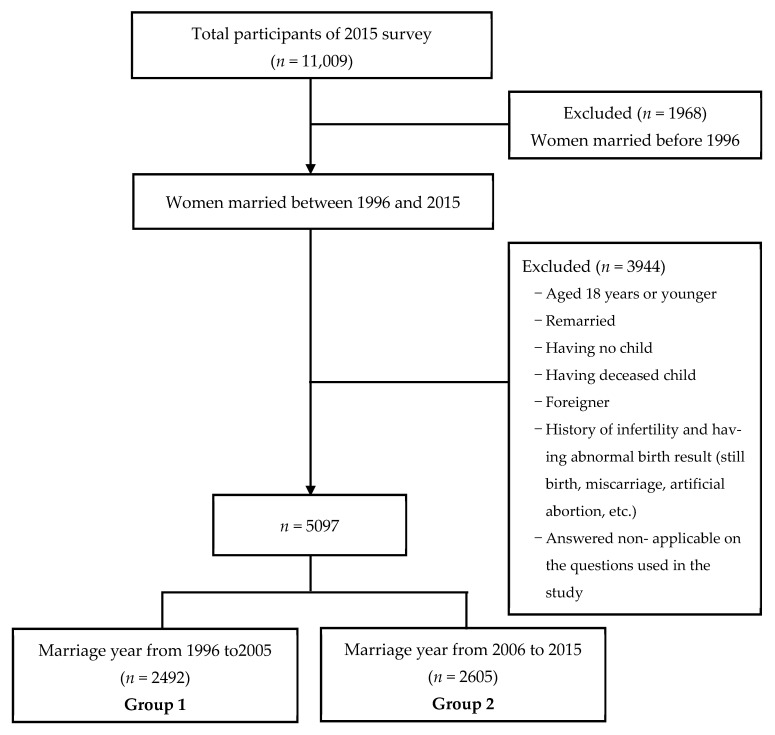
The selection process of participants.

Figure 2 shows the process of sample selection for this secondary data analysis. A total of 11,009 women participated in the 2015 NSFFHW. Among 11,009 total women participants, 1959 women married before 1996 were excluded. Additionally, 3944 women were excluded based on the exclusion criteria: (1) those who aged 18 years or younger because they legally need their parental consent for the marriage; (2) those who were remarried or foreigners; (3) those who had a history of having no children, child’s death, stillbirth, and infertility; and (4) those answering ‘non-applicable’ on the specific questions related to our study variables. After applying these exclusion criteria, a total of 5097 women remained and then they were divided into those married between from 1996 to 2005 (*n* = 2492) and those married between from 2006 to 2015 (*n* = 2605).

Participants, 1959 women married before 1996 were excluded. Additionally, 3944 women were excluded based on the exclusion criteria: (1) those who aged 18 years or younger because they legally need their parental consent for the marriage; (2) those who were remarried or foreigners; (3) those who had a history of having no child, child’s death, stillbirth, infertility; and (4) those answering ‘non-applicable’ on the specific questions related to our study variables. After applying these exclusion criteria, a total of 5097 women remained and then they were divided into those married between from 1996 to 2005 (*n* = 2492) and those married between from 2006 to 2015 (*n* = 2605).

### 2.3. Variables

#### 2.3.1. Dependent Variable

The dependent variable was the subsequent childbirth. It is a dichotomous variable showing whether there were any subsequent childbirths after the first one. Considering the question “Did you give birth to your second child normally?”, the women who answered it with “normal delivery” were categorized under “Yes”, whereas those pregnant with their second child or having one child at that time were categorized under “No”, respectively. The time to subsequent childbirth was determined as the period between the day of the first and the day of the second childbirth.

#### 2.3.2. Independent Variables

Demographic factors included current age, marriage age, age at the first birth, and education level. Age was a continuous variable, and educational levels were categorized into three groups as high school graduation or less, college graduate, and graduate school or above. Marriage age was divided into four groups: 19 to 24 years old, 25 to 29 years old, 30 to 34 years old, and 35 years old or older. Socio-economic factors included house ownership, house income, and maternal employment status. House ownership and maternal employment status were divided into “Yes” or “No”. House income was divided into four groups.

The value factors included son preference, child preference, and attitude toward the child. Both son and child preferences were assessed using the questions “Do you think you need to have a son?” and “Do you think you need a child?”, respectively. The answers consisted of “It must be”, “It will be better than nothing”, and “It does not matter”; women who answered on “It does not matter” were categorized “No” and others did “Yes”. Regarding attitudes toward their child, the mean score of six questions (“Being a parent is a worthwhile thing in life”; “A child makes a stronger relationship between husband and wife”; “People with children are less lonely in old age”; “If you have children, you can be helped economically in old age”; “It is natural to have children for generations of your family”; and “Having children is also a duty to society) were used [18], and each question was measured on a 4-point Likert-type scale (“entirely agree”, “mostly agree”, “mostly disagree,” or “ entirely disagree”). Higher scores indicate a more favorable attitude to having a child.

Support factors included housework fairness of husband and parents supporting for childcare. Housework fairness was measured using the question “Do you think your husband equally does the housework?” and parents support for childcare was assessed using the question “In the past 6 months, have you had economic or non-economic support for child care from your parents and/or parents-in-law?”. These variables were categorized into “Yes” or “No”.

### 2.4. Statistics

Descriptive statistics, including means, standard deviations (SD), frequencies, and percentages, were performed to examine the general characteristics and the variables related to childbirth. Discrete-time hazard models were performed to examine the factors associated with subsequent childbirth intervals from the first childbirth. This method is helpful in the analysis of the probability of an event occurring when the time variable is discretely measured. There was no evidence of multicollinearity between variables. All statistical analyses were performed using the SAS 9.4 (Cary, NC, USA) with a significance level of 0.05.

### 2.5. Ethical Consideration

This study was approved by the Institutional Review Board of Yonsei University in Korea (No. 4-2017-0876) to ensure the exempt status of the full review.

## 3. Results

### 3.1. General Characteristics of Participants by Groups

Table 1 presents the general characteristics of the participants in two groups by marriage year. The mean current and marriage ages of women were 40.39 years old (SD = 3.44) and 26.29 years old (SD = 2.78) in women married between 1996 to 2005 (Group 1); and 33.38 years old (SD = 4.03) and 28.52 years old (SD = 3.49) in women married between 2006 to 2015 (Group 2). The mean age at the first birth were approximately 26.61 (SD = 3.03) and 28.85 years (SD = 3.62) for the two groups, respectively. Approximately 60% and 75% of the women surveyed were at least college graduates in Group 1 and Group 2, respectively. Approximately 59% of women were employed in Group 1, and 41% were in Group 2. The mean number of children was 2.08 (SD = 0.61) in Group 1. In contrast to this, the mean number of children was 1.40 (SD = 0.82) in Group 2. Regarding son and child preferences, about 34% and 91% of women preferred to have a son and a child in both groups, respectively. About 45% and 1.77% of women reported as doing fairly housework with husband and having parents’ supports for their childcare, respectively, in Group 1; however, the rates of these items were 52.4% and 4.3%, respectively, in Group 2.

### 3.2. Differences in Factors Associated with Subsequent Childbirth between Two Groups

Table 2 presents the results of a discrete-time hazard models analysis regarding factors associated with subsequent childbirth in both groups. The common factors associated with the subsequent childbirth in both groups were maternal education level, having a son preference, and a positive attitude towards the child. Women with college graduation (hazard ratios, HR = 0.85; 95% CI = 0.77–0.93 in Group 1, HR = 0.89; 95% CI = 0.81–0.98 in Group 2) were less likely to have the subsequent child compared to women with high school graduation. Otherwise, having a son preference (HR = 1.16; 95% CI = 1.06–1.27 in Group 1, HR = 1.14; 95% CI = 1.04–1.24 in Group 2) and women with a higher score for a positive attitude toward the child (HR = 1.12; 95% CI = 1.01–1.25 in Group 1, HR = 1.11; 95% CI = 1.01–1.22 in Group 2) were more likely to have the subsequent child.

Regarding indifferent factors associated with subsequent childbirth between the two groups, there was the maternal age at the first childbirth (HR = 0.93; 95% CI = 0.91–0.95 in Group 1, HR = 1.35; 95% CI = 1.31–1.39 in Group 2). A monthly house income (USD) from 4600 to 6000 (HR = 1.18; 95% CI = 1.04–1.33) significantly increased the hazard ratios of subsequent childbirth rather than the reference group in Group 2 only. Working women (HR = 0.86; 95% CI = 0.78–0.94) significantly decreased the hazard ratios of subsequent childbirth compared to non-working women in Group 2.

## 4. Discussion

This study aimed to identify factors associated with subsequent childbirth in married women by marriage years. The major findings are as follows. First, maternal education level, which was a socio-economic factor, was associated with decreased subsequent childbirth in both groups. Second, values factors, such as son preference and positive attitude toward the child, were associated with increased subsequent childbirth in both groups. Third, only in Group 2 was higher monthly income associated with increasing subsequent childbirth; by contrast, women’s employment decreased the likelihood of subsequent childbirth. Finally, the variable that obtained contradictory results in the two groups was the age at first childbirth.

Firstly, the current study found that changes in fertility were associated with socioeconomic factors of women, such as education level. According to European studies, women with advanced education defined as tertiary education started a family later in life and postponed their first childbirth [8,19]. The association of advanced education with delayed marriage and childbirth can be understood as reflecting a need for women with advanced education to have more time to complete their education and also get established in a good job [20]. In Korea, according to 2017 statistics, the college enrollment rate of female students (72.7%) has steadily surpassed that of males (65.3%) since 2005 [5]. In other words, higher occupational and income opportunities for women with advanced education may create more significant conflict between their childbearing including subsequent childbirth and employment role [20]. In this context, regarding women’s employment, previous studies found that full-time working women were less likely to have subsequent child than were non-employed women [21]. Similarly, the current study found that employed women in Group 2 were less likely to have a subsequent child.

The Korean government has implemented plans for approaching low fertility and an increasingly aged society since 2006. The first plan (2006–2010) aimed at fostering environments favorable of childbirth and childcare and the second plan (2011–2015) aimed to foster a family-friendly and gender-equal culture.

Notwithstanding the efforts of the Korean government, including diversification of childcare leave, flexibility of labor conditions, and government support for women’s return to the labor market after completing early childcare, the index of work–life balance of Korea was 5.0, ranking the country third from the bottom among OECD countries [22]. The rate of flexible work in Korea including part-time work, staggered office hours, flexible working hours, and work from home was about 22%, which is very low compared with other developed countries [23]. In the U.S., 81% of companies have implemented a policy of staggered office hours, and 38% have implemented work from home [24]. In addition, 69% and 66% of European companies have adopted part-time work or staggered office hours, respectively [25]. It seems that women highly value work–life balance; thus, worksite or institutional support is a key factor in promoting childbirth, especially in promoting subsequent childbirth, among employed women. Previous cross-cultural research found that women prefer to housework more than men [26]; thus, housework may not be a harsh burden for all women. Actually, women provide more childcare than men in all cultures [27], including Korea. Women typically provide subsistence labor in tribal cultures. Therefore, societies must accommodate women’s labor as well as their being the main parent. Liberal paid parental leave, as well as other services, seem to ease child rearing and breastfeeding. The proportion of part-time job in Korean women is lower than that of OECD countries; however, it has been increasing since 2010 [28]. Furthermore, the rate of parental leave in Korea is 22.8 per 100 children, which is significantly lower than the lowest rate among OECD countries (female use rate: 53.3%). The actual period of use is an average of 7.7 months, and the biggest obstacle to the decision to take parental leave is economic difficulties [29]. For these reasons, many mothers prefer part-time labor because of parenting and financial concerns. In Europe, because of universal health coverage, part-time workers retain their medical benefits, whereas in the US, many young mothers must work full-time in order to retain coverage. Therefore, institutional arrangements should be supplemented to ensure flexible and stable work.

Second, age at the first birth was associated with fertility. Due to the limited childbirth period, women are less likely to have children if their first birth is delayed. According to Korean statistics, women’s age at first marriage was higher in the more recent period of marriage [30]. In detail, the average first marriage ages increased from 25.8 years in the period between 1995–1999 to 29.9 years in the period between 2010–2015. Along with this demographic trend, women’s mean age at first birth in Korea had increased from 26.7 years in 1996 to 31.2 years in 2015 [30]. It is noteworthy that, in Group 1, before implementing the childcare policies, the occurrence of subsequent childbirth decreased as the age at first birth increased. In contrast, in Group 2, during the years of implementation of childcare policies in Korea, the hazard ratio for subsequent childbirth increased with the age at first birth. According to Korean statistics, the total number of childbirths with subsequent childbirth showed a downward curve until 2005, before representing an upward trend after 2006. In detail, the number of subsequent childbirths increased from 166,900 in 2005 to 184,000 babies in 2012 [31].

The government unveiled the first plan of a mid-term childcare plan for the period 2006 to 2010, known as the “Saessak Plan” in 2006. The Saessak in English means “newly sprouted shoots”, which is often used to refer to children in Korea. The purpose of these policies was to strengthen the publicity of childcare and provide high-quality childcare services. The Korean government has steadily increased the budget for childcare support and undertaken the following childcare support projects: improving access to childcare facilities (e.g., daycare centers, kindergarten), reducing household burden due to childcare expenses, and enhancing the quality of childcare services. As a result, the number of childcare facilities increased from 19,276 in 2000 to 35,352 in 2020, and the childcare fee has been fully subsidized except for some of the upper-income households since 2012 in Korea [32]. It seems that these aggressive government policies appear to have had an impact on the fertility rate to some extent.

Third, the value of sex preference has an impact on reproductive behavior. The current study shows that women who prefer a son were more likely to have a subsequent child than women who did not. This pattern has been found in some Southeast Asia, including Vietnam, China, and India [33]. The fundamental idea is that couples have a strong preference for sons, and that they are more likely to have an additional child if they have an only daughter as a first baby or if there are no other restrictions (e.g., economic problems, maternal age). Sons tend to be perceived as significant economic resources of the household in the form of a patriarch in the modern society of Southeast Asia [34]. Regarding positive perceptions about the VOC, they were also more likely to give birth to a child. Having a child is the result of a complicated rational decision. Ajzen’s study (2013) using the Theory of Planned Behavior [35] found that attitudes towards having a subsequent child had the most substantial effect on childbearing intentions [36]. Ajzen’s study [36] reported that the decision to have their subsequent child seems to be mainly related to their beliefs based on the idea that having another child will make their lives better or worse. In this context, the experience of raising the first child can be important and can thus improve the parenting environment to relieve parents’ burden to raise a child will be needed for encouraging childbirth. Furthermore, a positive perception on having a child to be a source of happiness would compensate the burdens of childcare. Positive parenting experience and environment are highly likely to act as important factors for realizing the importance of child existence for both the mother and child [37].

This study has several limitations. This study used cross-sectional data, and, thus, causal relationships among the variables examined cannot be determined. Therefore, further studies using a longitudinal design to examine the causal relationship of the variables are needed. Another methodological concern limited the consideration of a wide range and selection of variables due to the study design focusing on secondary data analysis. For example, both mother’s and father’s employment is important to having children; however, this study did not include father’s employment status. Work types among women which may affect childbirth were not included in the analysis. In detail, several studies had shown that women with a secure job [8,38,39] or a job that guarantees a work-life balance [8] were more likely to be associated with subsequent childbirth. It included only the two questions about the point of view on son preference and the value on economic and social utility. Future research should include such an item and other more precise questions regarding positive perception towards their child. Finally, support factors, including housework fairness and parental support in childcare, were not statistically significant in this study. Cross-cultural research indicates that help provided by other relatives can ease the mother’s burden, and presumably making her more willing to have subsequent children [15]. The father’s assistance tends to improve the wife’s marital satisfaction [40]. Grandparents typically help out with child care in our species. Older siblings, especially daughters, typically contribute care, actually enhancing the mother’s health and longevity [41]. Societal measures to raise the birth rate are more likely to succeed if they accommodate human nature as indicated by cross-cultural research patterns. Despite the presence of various influencing factors as in previous studies related to social support, there was a limitation for using variables on supportiveness resources, such as the husband’s and parents’ factors for childcare in the current study. In detail, the questions related to childcare support consisted of whether respondents received financial or non-financial support from their parent-in-laws or their parents in the past 6 months. In addition, there was limited use of appropriate questions regarding the husband’s contribution to domestic work. Thus, further primary data collection should be conducted with more valid questionnaires consisting of multidimensional components, including cultural issues, specifically regarding parental support for childcare.

## 5. Conclusions

This study shows that changes in demographic characteristics, such as delayed marriage, higher education attainment, and employment, result in declining fertility rates in Korea. To increase fertility in Korea, the government needs to more actively make provisions for childcare and deal with the reduction in the incomparable role for women under the circumstance of changes in demographic characteristics.

## Figures and Tables

**Table 1:** General characteristics of participants.

Variables	Group 1 ^a^ (*n* = 2492)	Group 2 ^b^ (*n* = 2605)
	Mean ± SD (Range) or *n* (%)	Mean ± SD (Range) or *n* (%)
Current age (year)	40.39 ± 3.44 (29–49)	33.38 ± 4.03 (19–48)
Marriage age	26.29 ± 2.78 (19–38)	28.52 ± 3.49 (19–44)
<25	610 (24.48)	268 (10.29)
25–29	1590 (63.80)	1433 (55.01)
30–34	277 (11.12)	764 (29.33)
≥35	15 (0.6)	140 (5.37)
Age at the first birth	26.61 ± 3.03 (18–44)	28.85 ± 3.62 (18–44)
Educational level		
High school graduation	975 (39.13)	662 (25.41)
College graduation	1422 (57.06)	1806 (69.33)
≥Graduate school	95 (3.81)	137 (5.26)
House ownership		
No	700 (28.09)	1248 (47.91)
Yes	1792 (71.91)	1357 (52.09)
House income (USD/month)		
≤3500	508 (20.39)	921 (35.36)
3500–4600	701 (28.13)	710 (27.26)
4600–6000	617 (24.76)	518 (19.88)
≥6000	666 (26.73)	456 (17.50)
Maternal employment		
Employment	1470 (58.99)	1073 (41.19)
Unemployment	1022 (41.01)	1532 (58.81)
Number of children	2.08 ± 0.61 (0–10)	1.40 ± 0.82 (0–4)
1	290 (11.64)	1144 (43.92)
2	1750 (70.22)	1268 (48.68)
≥3	452 (18.14)	193 (7.40)
Son preference		
No	1632 (65.49)	1733 (66.53)
Yes	860 (34.51)	872 (33.47)
Child preference		
No	201 (8.07)	223 (8.56)
Yes	2291 (91.93)	2382 (91.44)
Attitude toward child (Range: 1–4)	2.83 ± 0.43	2.82 ± 0.44
Housework fairness		
No	1353 (54.29)	1239 (47.56)
Yes	1139 (45.71)	1366 (52.44)
Parental support in childcare		
No	2448 (98.23)	2494 (95.74)
Yes	44 (1.77)	111 (4.26)

^a^ Group 1: women who married between 1996 to 2005; ^b^ Group 2: women who married between 2006 to 2015.

**Table 2:** Factors associated with subsequent childbirth by groups of marriage year (1996–2005 vs. 2006–2015).

	Variables	Categories	Group 1 ^a^ (*n* = 2492)			Group 2 ^b^ (*n* = 2605)		
			HR	95% CI		HR	95% CI	
Demographic Factors’	Current age (year)	N/A	1.02	1.01	1.04	0.72	0.70	0.74
	Marriage age (ref. = 25–29)	<25	0.84	0.73	0.96	0.96	0.81	1.14
		30-34	1.16	0.97	1.37	1.13	1.00	1.28
		≥35	1.07	0.55	2.07	0.90	0.68	1.19
	Age at the first childbirth	N/A	0.93	0.91	0.95	1.35	1.31	1.39
Socio- Economic Factors	Education level (ref. = High school Graduation)	College graduation	0.85	0.77	0.93	0.89	0.81	0.98
		≥Graduate school	0.85	0.67	1.09	0.79	0.65	0.97
	House ownership (ref. = No)	Yes	1.09	0.99	1.21	0.92	0.85	1.00
	Monthly house income, USD (ref. = <3500)	≥3500, <4500	1.02	0.90	1.16	1.10	0.99	1.23
		≥4600, <6000	1.00	0.87	1.14	1.18	1.04	1.33
		≥6000	1.00	0.87	1.14	1.15	1.00	1.32
	Maternal employment (ref. = No)	Yes	1.04	0.95	1.14	0.86	0.78	0.94
Values Factors	Son preference (ref. = No)	Yes	1.16	1.06	1.27	1.14	1.04	1.24
	Child preference (ref. = No)	Yes	1.15	0.98	1.36	1.15	0.99	1.33
	Attitude toward child	N/A	1.12	1.01	1.25	1.11	1.01	1.22
Social Support Factors	Housework Fairness (ref. = No)	Yes	0.96	0.88	1.05	0.98	0.90	1.06
	Parental support in childcare (ref. = No)	Yes	1.14	0.83	1.57	1.01	0.83	1.24

ref. = reference; ^a^ Group 1: women who married between 1996 to 2005; ^b^ Group 2: women who married between 2006 to 2015.

## Data Availability

Data used for the secondary analysis in this study is available upon request. https://data.kihasa.re.kr/micro/request/list.jsp (in Korean) (accessed on 18 February 2018).
